# Forging a Bayesian link between habitat selection and avoidance behavior in a grassland grouse

**DOI:** 10.1038/s41598-021-82500-0

**Published:** 2021-02-02

**Authors:** Michael A. Patten, Alexandra A. Barnard, Claire M. Curry, Henry Dang, Rebecca W. Loraamm

**Affiliations:** 1 Ecology Research Group, Faculty of Biosciences and Aquaculture, Nord University, 7729 Steinkjer, Norway; 2 Oklahoma Biological Survey, University of Oklahoma, Norman, OK 73019 USA; 3 Math Sciences Division, National Park College, Hot Springs, AR 71913 USA; 4 University Libraries, University of Oklahoma, Norman, OK 73019 USA; 5 Department of Geography and Environmental Sustainability, University of Oklahoma, Norman, OK 73019 USA

**Keywords:** Behavioural ecology, Conservation biology, Grassland ecology

## Abstract

Habitat selection is a basic aspect of the ecology of many species, yet often the term is conflated or confused with both habitat preference and habitat use. We argue that each term fits within a conceptual framework that can be viewed in Bayesian terms and demonstrate, using long-term data on occupancy patterns of a grassland grouse, how prior probability profiles can be estimated. We obtained estimates by specifically focusing on whether and to what extent the Lesser Prairie-Chicken (*Tympanuchus pallidicinctus*) avoids anthropogenic features such as roads, powerlines, petroleum wells, fences, and buildings, in two study areas, one with denser and one with sparser incidence of features. Grouse strongly avoided large features such as outbuildings and tended to avoid tall features such as powerlines; by contrast, grouse did not or only slightly avoided low, unobtrusive features such as fences. We further examined co-location of pairs of anthropogenic features and found that certain features were avoided so strongly that avoidance distance may be shorter for other features; that is, birds were “pushed toward” some features because they are “pushed away” from others. In each case, our approach points toward a means to incorporate avoidance behavior directly into analytic studies of habitat selection, in that data on use (the posterior, as it were) could be used to infer the selection process provided data on preference (the prior, as it were) could be obtained.

## Introduction

**Figure 1:**
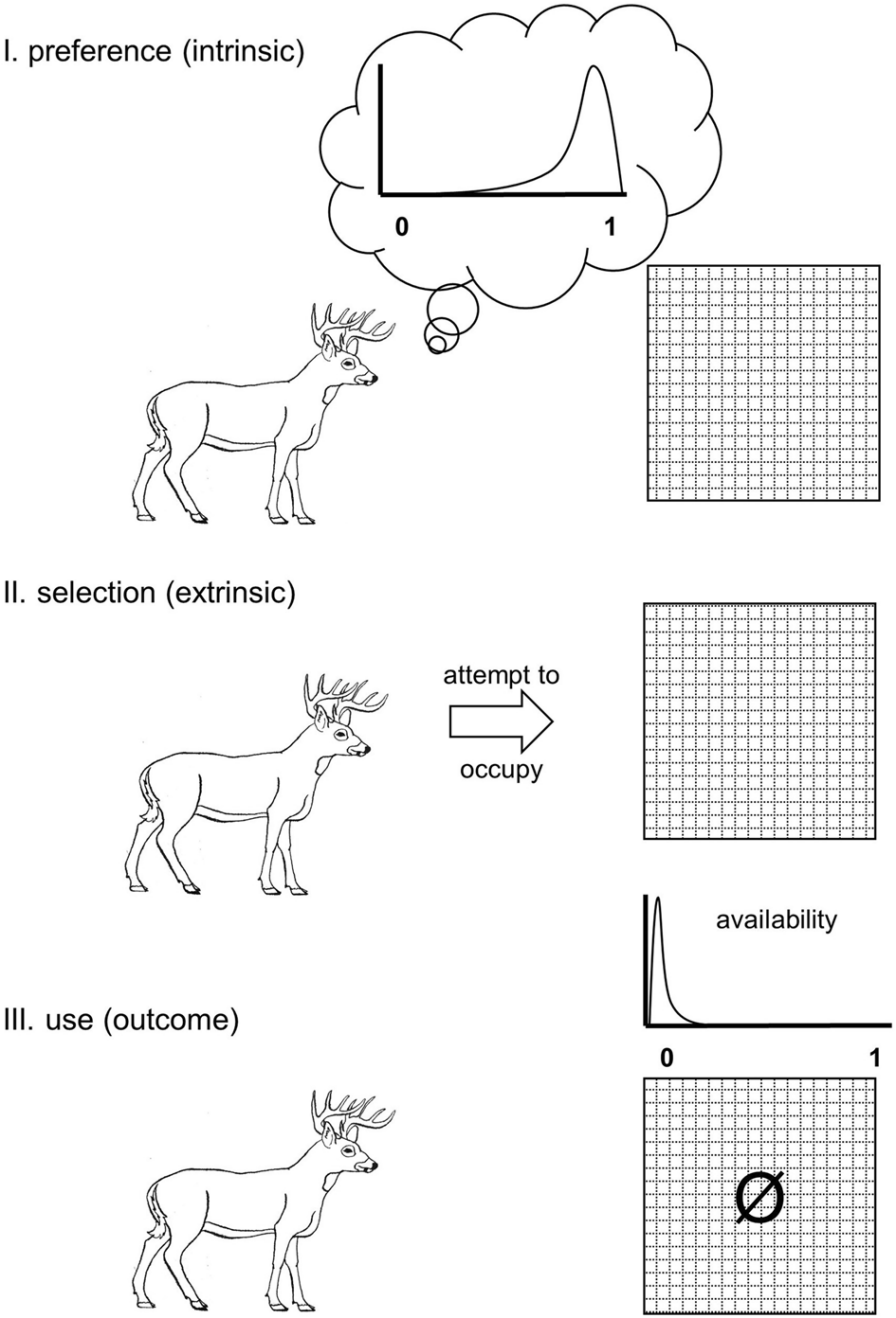
Conceptual schematic of distinctions and relationships between habitat preference (here with an example of a probability distribution for a single cue, i.e., landscape feature), habitat selection, and habitat use. For the example of use, availability is considered to be near zero, so the patch (the hatched box) is unoccupied.

Habitat selection, preference, and use are not equivalent terms. *Selection* refers to non-random occupancy of habitat bearing some consequences on individual fitness^1^, *preference* is conceptualized as a probability distribution of an individual’s selection for a habitat when offered alternatives^2^, and *use* describes resultant patterns, which may be mitigated by factors outside of preference, such as competition pressure or predation risk. Habitat selection itself is active, not passive, with a prerequisite of cognitive assessment among available choices (Fig. 1).

**Figure 2:**
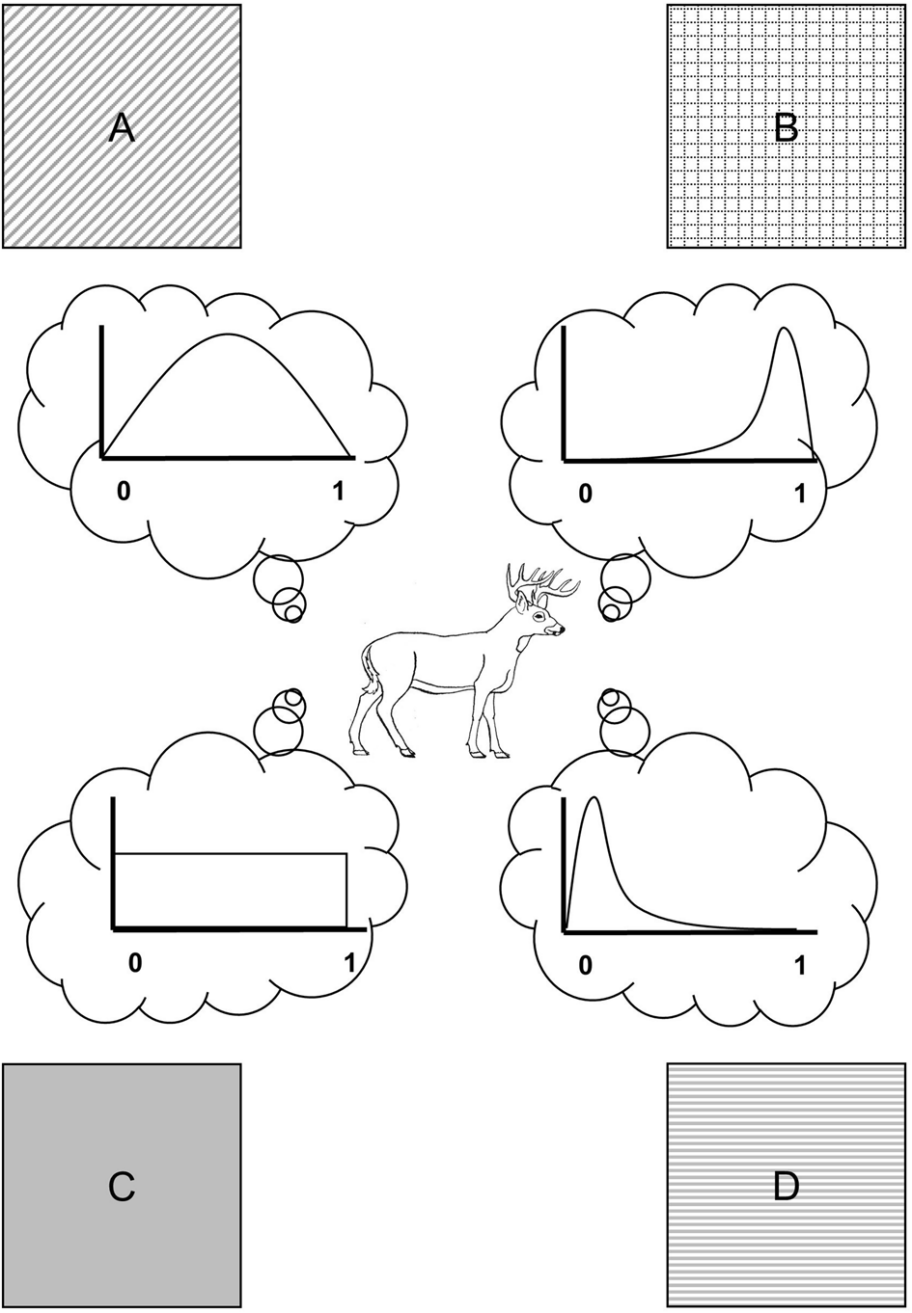
Conceptual schematic of habitat preference viewed as a probability distribution. Four habitats, (**A**–**D**) are available to the organism, which has a moderate preference for (**A**), strong preference for (**B**), low preference for (**D**), and no preference for (**C**). We posit that such distributions can be used as priors in a Bayesian framework.

A key aspect of cognitive assessment inherent to habitat selection is the possibility of *avoidance*, a type of assessment that has its own consequences^3^. Avoidance is both an outcome of intrinsic negative preference and a complex reaction attenuated by an individual's ecological context. Even so, avoidance is not merely an individual's maintenance of negative preference for a given habitat, even though patterns of use may appear equivalent in a binary case. For example, if an organism prefers habitat B to habitat A (Fig. 2), it may select habitat B every time when only A and B are available, but it may not avoid habitat A in another context, as when the second option is habitat D. In that case, the organism may select habitat A every time given those two options but always avoid habitat D regardless of the alternative, even against an option for which an individual has no preference (habitat C in Fig. 2).

It is tempting, then, to conclude that preference and subsequent selection are relative, with preference expected to be “hard wired” to the extent it is under natural selection^4^, meaning some aspect of individuals’ habitat selection will reflect the landscape’s contextual history^5^. In this view, an organism will assess particular cues, such as vegetation structure or water availability (among many possibilities), to build its probability distribution of preference. Human alteration of the landscape’s fragmentation, biotic interactions, and physical structure (our focus in this study) can disconnect cues from outcomes (i.e., fitness components) either by leaving cues unchanged but reducing fitness (ecological traps^6^) or leaving fitness unchanged but altering cues (perceptual traps^3^). Potential fitness consequences have received the bulk of attention in the literature, including the role of habitat avoidance in speciation^7^, yet altered cues matter a great deal given that otherwise suitable habitat may be avoided.

Habitat avoidance falls under the purview of sensory ecology, “the study of how organisms acquire and respond to information in their environment”^8^, with avoidance comprising an outcome of “acquisition of information from the abiotic environment (*cues*) by a single organism”^8^ and subsequent processing of that information. Estimating the extent and magnitude of avoidance is a challenge precisely because an organism will either not occur in or occur in low densities in habitats perceived to be unsuitable (e.g., habitat D in Fig. 2). Here avoidance must be identified by absence of certain habitat cues, which assumes sufficient knowledge of a species’ ecology, such as consistent presence of cues in occupied habitat. Despite this complication, we can make substantial inroads toward understanding what information matters to an individual’s avoidance with rigorous evaluation of patterns of habitat use and exploration of cues that vary across the landscape.

We suggest a conceptual approach to model and analyze the overall process grounded in Bayesian thinking. Frequentist statistics test how well data fit an established model (e.g., a *t* distribution); Bayesian statistics test how well models fit data at hand and explicitly take into account prior knowledge of data distributions or parameter estimates. We posit that preference can be treated as an informative prior, a statistical distribution of preference grounded in existing knowledge of a species’ habitat preferences and habitat selection cues. It is standard in Bayesian models to update the prior (i.e., a parameter estimate) with subsequent information. Our approach resembles the concept that habitat preference, selection, and use form a feedback loop resulting from individual behavior across generations. Treating preference as a prior suggests a direct utility of analyses of avoidance behavior such as ours, in that results could be used to build a probability distribution across selected cues rather than beginning with an assumption of flat priors, an assumption that animals have no initial preference (e.g., Fig. 2C). We further posit that selection, as we restrict its definition (Fig. 1), can be treated as the likelihood. It is the process that, combined with the prior, determines extrinsic behavior. The endpoint of such a Bayesian formulation is a posterior distribution (the model estimate) that can be viewed as use (Fig. 1).

We use long-term tracking data in an anthropogenically modified landscape to assess patterns of avoidance and four potential causal processes of it. In our system, cues were altered in that humans placed objects on a landscape, objects such as powerlines, roads, or oil wells. On the basis of research on avoidance behavior in grouse^9,10^, it is expected such objects (hereafter called features) would affect the sensory ecology of our focal species, the Lesser Prairie-Chicken (*Tympanuchus pallidicinctus*), a rare and declining grouse endemic to arid grasslands of the south-central United States (see “Focal species”, below). We postulated four novel hypotheses that explain how and why individual grouse avoid these man-made features.

1. “Proportional use” posits that grouse will, on average, distribute themselves across the landscape at distances directly in proportion to the spatial density of any anthropogenic feature. In essence, then, this idea corresponds to a null expectation and as such implies no avoidance.
2. “Egalitarian avoidance” posits demonstrable equitable non-use of areas near to any anthropogenic feature; i.e., all anthropogenic features are avoided equally. Under this hypothesis, avoidance distance from feature A is the same as it is to feature B, regardless of the spatial density of either feature.
3. “Selective avoidance” differs from “egalitarian avoidance” in that grouse avoid some features more than others. Perhaps they avoid roads more (i.e., avoidance at a greater distance) than fences because the former is noisier, or perhaps they avoid powerlines or outbuildings more than oil or gas wells because these features are more prominent (both taller and seemingly more massive). As with the first two hypotheses, “selective avoidance” does not consider spatial density of a given feature.
4. “Rather be elsewhere” posits that apparent avoidance of a feature reflects either a preference for another feature or a density-dependent—in terms of the features, not of the grouse—response such that stronger avoidance of feature A “pushes” a grouse nearer to feature B, which it otherwise would avoid but cannot given its situation.

Grouse that avoid a feature ought to occur farther (statistically) from a feature than realizations of random points would in the same context. A corollary to this simple decision rule is that statistically nearer distances could be considered preference for proximity to a feature. This rule and associated analyses led us to key statistical predictions for a distance parameter, d[], for each hypothesis with regard to random points (R) and grouse locations (G):

- H_1_: no difference; i.e., d[R] = d[G].
- H_2_: d[G] > d[R], with d[G_*i*_] = d[G_*j*_] for any feature *i* or *j.*
- H_3_: d[G] > d[R], with d[G_*i*_] > d[G_*j*_] for some features *i* and *j*, *i* ≠ *j.*
- H_4_: d[G_*ij*_]/d[R_*ij*_] < 1, = 1, or > 1 if, respectively, nearer to, the same distance from, or farther from feature *i* versus feature *j*, *i* ≠ *j*, for grouse occurrences relative to random points.

The final prediction indicates whether bird occurrences are clustered, randomly placed, or dispersed relative to random points for any two features (i.e., covariation in feature avoidance). We tested each prediction with a suite of Bayesian models.

## Methods

### Focal species

The Lesser Prairie-Chicken is a medium-sized grouse endemic to, broadly, the shortgrass prairie ecosystem of the south-central United States, where it is found only in southwestern Colorado, western Kansas, northwestern Oklahoma, the Texas panhandle, and eastern New Mexico. As with almost all open-country grouse of temperate environments, the Lesser Prairie-Chicken forms leks at which a cluster of males (in this species, usually 5–12 individuals) display vigorously and female visit to assess males with an intent to secure sperm to fertilize her eggs, which she will lay and raise without male help. Outside of male lekking (mid-March to mid-May) and female nesting (late April to early July), birds congregate is small flocks to forage, at times on grain remains in farmed fields but typically, as through the rest of the life cycle, restricting themselves to native prairie. Accordingly, this species has three distinct aspects of habitat selection: general occurrence, lek placement, and nest placement.

### Data

Lesser Prairie-Chicken were tracked at two study sites, one in Roosevelt County in east-central New Mexico, U.S.A., the other in Beaver, Harper, and Ellis Counties in northwestern Oklahoma, U.S.A. Birds tagged with VHF transmitters were tracked from April 1999–March 2006 in New Mexico and from March 1999–July 2013 in Oklahoma^11–14^. Study periods differed chiefly because of funding, which for New Mexico was insufficient after 2006. The study sites differ markedly in land tenure history. Parcel size in New Mexico averages 1300 ha versus 180 ha in Oklahoma^11^. The difference largely stems from settlement patterns over the past two centuries. New Mexico was part of the Spanish land grant system, which tended to yield huge parcels. In our study area, parcels approach 8 km^2^ (> 1900 acres). By contrast, during the “land rush” era of the late 19th Century most of northwestern Oklahoma was parceled into 65-ha (160-acre) plots as part of the United States’ Homestead Act. Smaller parcels translate to a higher density of roads, fences, buildings, and powerlines^11^.

Radiotracking typically yielded a triplet of coordinate readings, from which we had to triangulate a grouse’s location. We estimated latitude and longitude using a maximum likelihood estimator (MLE), although it some cases the MLE algorithm failed to converge. If it failed, we instead used the Andrew and Huber methods^15^. R code for the estimation procedure can be found at https://github.com/henry-dang/triangulation/blob/master/lenth_triang.R.

From these data we used kernel density methods (R package *ks*^16^) to estimate annual home ranges (235 in New Mexico, 263 in Oklahoma). Tracking data included lek (12 in New Mexico, 23 in Oklahoma) and nest (122 in New Mexico, 128 Oklahoma) locations. For home range centroids, the outer contour of home ranges, leks, and nests, we estimated distance to seven anthropogenic features: roads (highways, primary, and secondary roads only; small farm roads or one-lane gravel roads were excluded), powerlines (overhead only, with buried or trunk lines excluded; https://hifld-geoplatform.opendata.arcgis.com/datasets/electric-power-transmission-lines), oil wells, gas wells (for both types of wells, http://www.occeweb.com and http://www.emnrd.state.nm.us/ocd), outbuildings (barns, grain silos, poultry houses, and similar large structures; chiefly the TIGER database), and fences (Bureau of Land Management) in both states, plus private houses in New Mexico and railroad tracks in Oklahoma. We placed 2000 random points on each study area to estimate distances to each of these same anthropogenic features, which provided an estimate of feature density on the landscape.

### Analyses

**Figure 3:**
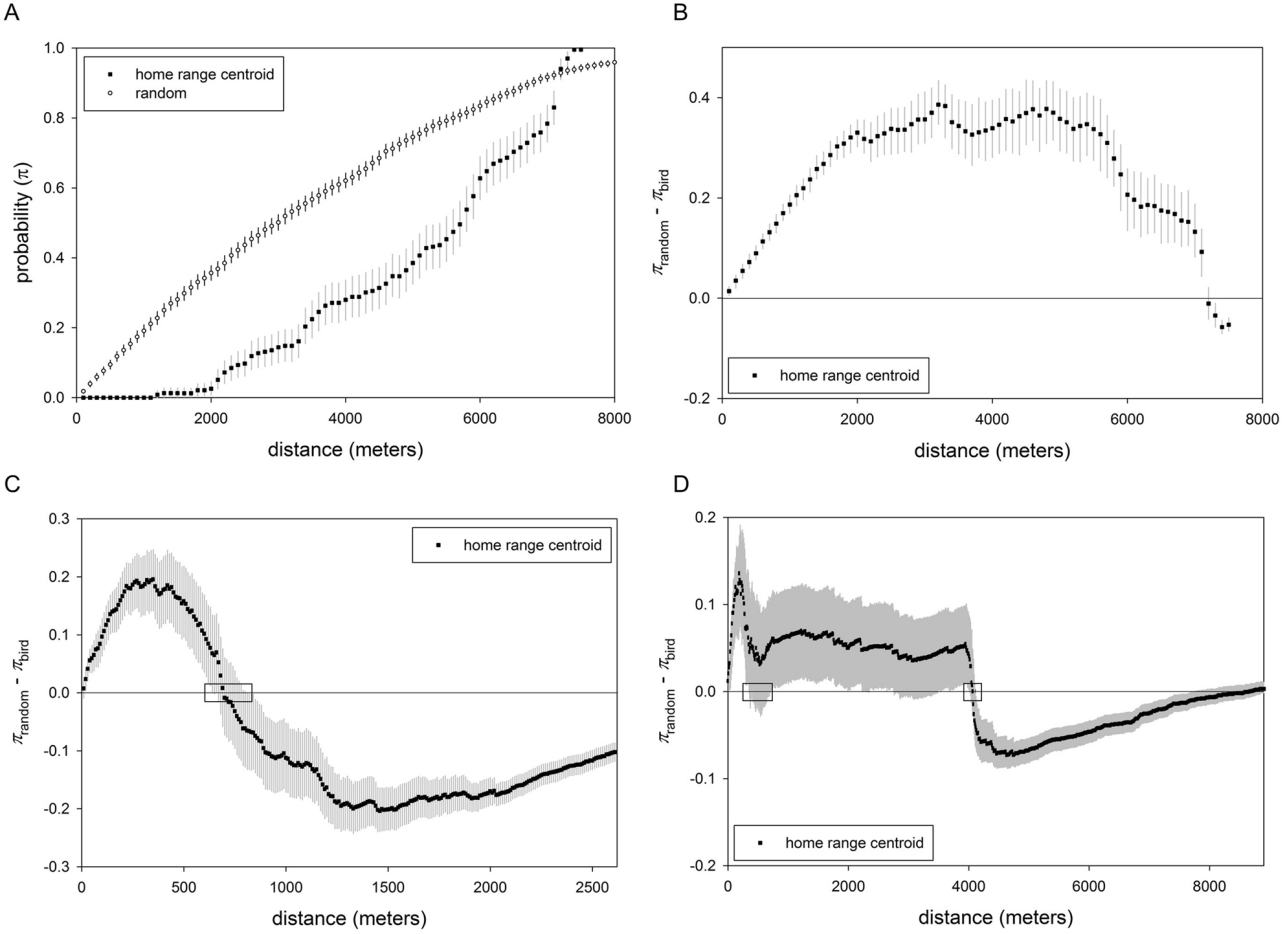
Analytic process used to determine presence and magnitude of avoidance thresholds. All examples use data for home range centroids. (**A**) Probabilities (± Bayesian highest density credible intervals) of occurrence of an individual Lesser Prairie-Chickens and random points at a given distance from an feature (in this case roads in New Mexico, binned at 100 m). (**B**) Difference in random and prairie-chicken points for the same data set. The point at which the difference curve crosses 0 is the threshold. (**C**) The search window (small box) to estimate the threshold often was unambiguous (fences, New Mexico, binned at 10 m). (**D**) For unclear cases, we conservatively searched for the threshold at the shorter distance (left box, where credible intervals cross 0) rather than the direct point (right box) crossed (fences, Oklahoma, binned at 10 m).

The initial step was to estimate the probability of a grouse occurring a certain distance from a feature. We treated New Mexico and Oklahoma data separately, giving us a replicate assessment because these populations have been isolated from each other for > 100 years^17^ and, as noted above, land tenure history differs strikingly between the states^11^. We estimated probabilities of grouse occurrence, *π*_*i*_, via a Bayesian model with binomial likelihood and flat prior (i.e., no assumption of the central tendency of occurrence probability at a given distance from a feature):

*y*_*i*_ ~ binomial (*π*_*i*_, *n*) with *y*_*i*_ the cumulative count of grouse at distance *i* (i.e., the data) and *n* the total number of home ranges, leks, or nests. Distances, *i*, were binned to the smallest extent possible, from 10 to 100 m, to allow the Markov chain Monte Carlo (MCMC) algorithm to converge in a reasonable number of iterations (e.g., < 100,000). (In Bayesian statistics, MCMC is used to build a posterior probability distribution from the product of the prior and the likelihood without having to integrate the often intractable denominator in the Bayes’ theorem formulation. The algorithm “allows one to characterize a distribution without knowing all of the distribution’s mathematical properties by randomly sampling values out of the distribution”^18^.) We used the same process for random points to estimate probability of feature occurrence at a given distance, the idea being to establish a null expectation of density of class in each study area. We plotted resultant bird and random curves to get an idea of whether grouse avoided the feature (Fig. 3A). If grouse neither avoided nor preferred a feature, then its curve and the random curve would trace similar arcs (within credible intervals; see below). If instead grouse avoided a feature, then its curves would intersect the random one at some distance from the origin (Fig. 3A); the converse holds for preference.

To assess if and to what extent grouse avoid a feature, we extended the Bayesian model to calculate a difference in probabilities between random points and grouse occurrence (Fig. 3B^19^). (At root, our method resembles a resource selection ratio^20^, but it was derived independently within a Bayesian framework.) We used the posterior probability distributions of these differences to search for the point at which grouse and random curves converged, the threshold distance (*T*). Our algorithm was.

1. calculate differences per bin, *π*_*R*i_–*π*_*Gi*_, with associated posteriors;
2. use the extent (*q*) to which a posterior distribution overlapped 0 to estimate the probability (*p*) that the threshold occurred in bin *k*, with *p*_*k*_ = 1 − 2|*q*_*k *_− ½|;
3. locate the apparent crossover region given calculated differences; usually the crossover was unambiguous (Fig. 3C), but if credible intervals overlapped 0 before the bulk of the distribution crossed (Fig. 3D), we conservatively used the region with the shorter distance so as not to overestimate distance;
4. estimate *T* as a weighted average of probabilities *p*_*k*_ ± 500 m of apparent crossover, with *p*_*k*_ as weights and for the *k*th bin (small boxes in Fig. 3C,D).

This algorithm yielded an estimate of *T* as a posterior probability distribution, from which we calculated threshold distance as the median and uncertainty as the highest density credible intervals, the broadest probability density that includes the mode. (A Bayesian credible interval is calculated directly from the posterior distribution and states that, say, 95% of parameter estimates lie between upper and lower bounds.) If the lower interval overlapped or closely approached 0, we could reasonably conclude the feature was not avoided. We supplemented threshold analyses with a Bayesian estimate, with log-normal likelihood and flat prior, of prediction intervals for grouse occurrence relative to a feature. From these estimates we calculated a coefficient of variation to evaluate whether areas with higher feature density had greater uncertainty for distance estimates. Lastly, we compared feature density at each study site by means of a Bayesian analog to a two-sample *t*-test^21^, with *t* likelihood and flat prior.

**Figure 4:**
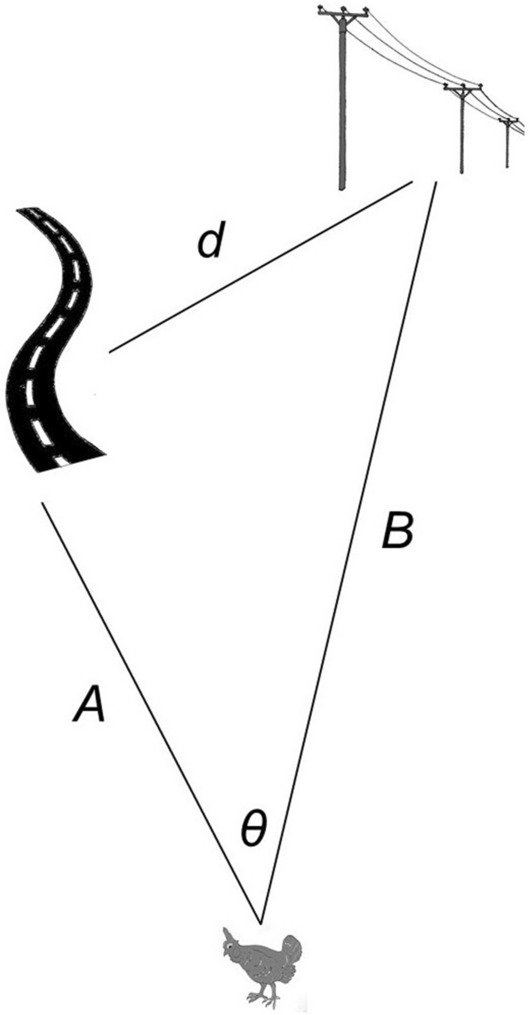
Schematic of metrics used to estimate distance between anthropogenic features.

Our final analysis assessed the extent to which avoidance of one feature might suggest preference for another or an instance of “co-avoidance”; i.e., it assesses colocation in a manner related to a cross-*K* function^22^. Available data were distance from a grouse (home range centroid only; sample size was otherwise too low) or random point to a given feature. We estimated distance, *d*, between one feature and another feature by means of the cosine rule:

$$d = \sqrt {A^{2} + B^{2} - 2AB\cos (\theta )}$$

where *A* is the distance from a grouse to the first feature, *B* is the distance from a grouse to the second feature, and *θ* is the angle between them (Fig. 4). We built a Bayesian model to estimate *d* for grouse (*d*_*G*_) and for random points (*d*_*R*_), with log-normal likelihood and *θ* as a flat prior. We used estimated distances to estimate a dispersion ratio, *d*_*G*_/*d*_*R*_, for which values less than one (i.e., below the lower credible interval of the estimate) implied a clumped distribution, values greater than one (i.e., beyond the upper credible interval) implied a dispersed distribution, and values between (i.e., within the credible intervals) implied a random distribution. The logic behind this ratio is that if avoidance of one feature was stronger than avoidance of another, then the ratio would increase: if *B* is unchanged, a decrease in *A* will increase *d* (Fig. 4), implying that strong aversion of *B* “pushes” organisms nearer to *A* as an effect of their preferences. The reverse situation implies that an organism is equally averse to the features or prefers one feature to another even if both are avoided (i.e., they are “lesser evils”).

Each of our Bayesian models was coded in JAGS and run via package *rjags*^23^ in R, with associated R code to support hierarchical flow (i.e., to manage data between some stages of the analyses).

## Results

Density of all features across the landscape was markedly higher in Oklahoma than in New Mexico (Table 1). Even in the case of road density, for which the 95% highest density credible intervals of the difference overlapped 0, it remained that with probability 0.93 the true and underlying mean distance to a road from a random point in New Mexico was farther than a random point in Oklahoma.

**Table 1:** Distribution across the landscape in New Mexico (NM) versus Oklahoma (OK) of various man-made features.

Feature	NM	OK	Difference (HDI)	Proportion > 0 (< 0)	η
Road	3362	3252	110 (− 36, 256)	0.928 (0.072)	0.047
Powerline	18,105	5778	12,327 (11,928, 12,734)	1.000 (0.000)	1.940
Oil well	8117	5480	2637 (2331, 2928)	1.000 (0.000)	0.552
Gas well	8387	695	7692 (7438, 7950)	1.000 (0.000)	2.521
Outbuilding	12,438	5591	6847 (6593, 7093)	1.000 (0.000)	1.712
Fence	457	234	223 (188, 260)	1.000 (0.000)	0.656

Results are from a Bayesian comparison of means (i.e., an analog to a two-sample ANOVA or *t*-test) of distance (m) to feature of 2000 random points placed in each study area. HDI refers to the Bayesian highest density credible interval, proportion > 0 refers to the proportion of the posterior distribution positive (or negative), and η is the effect size.

**Figure 5:**
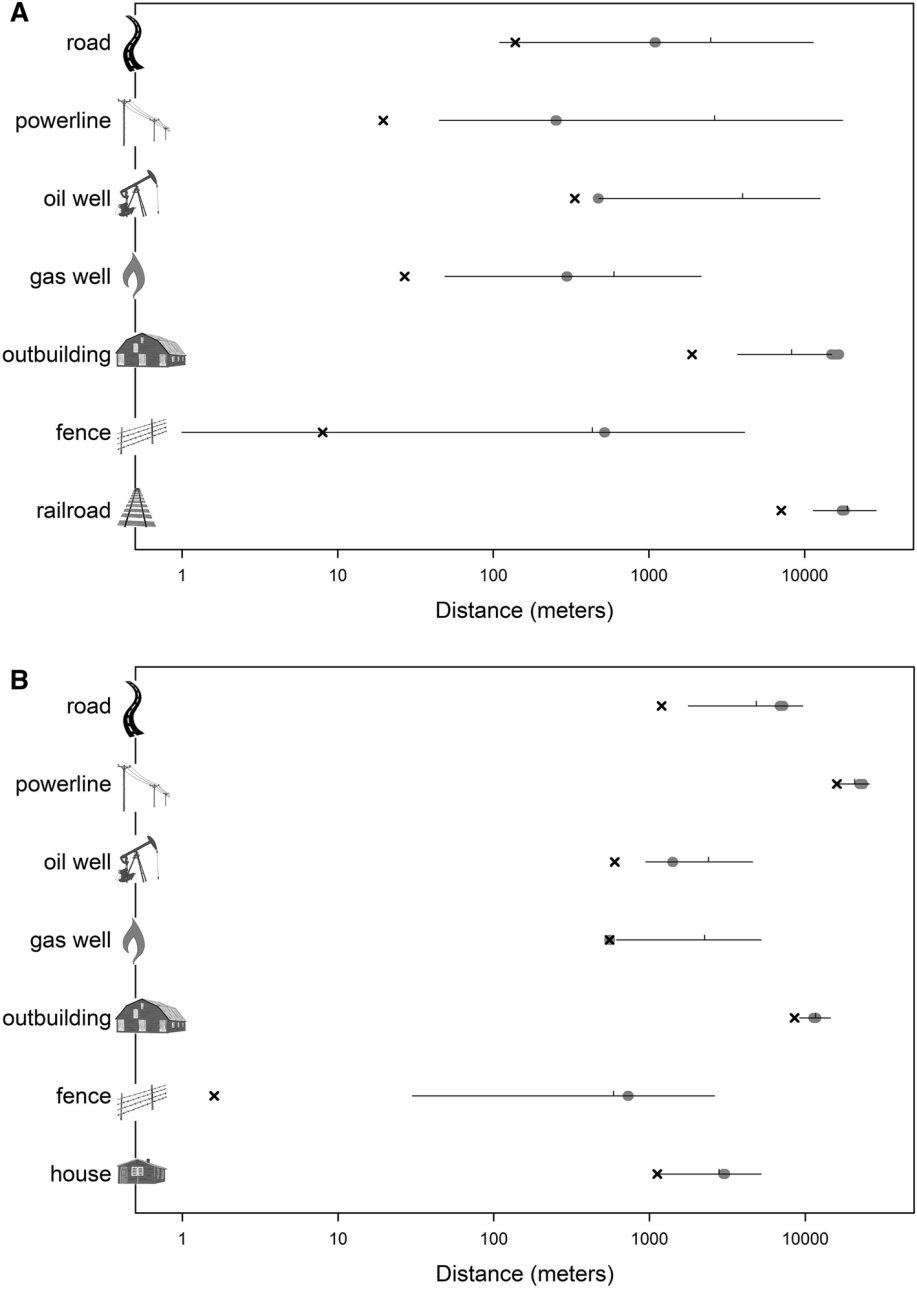
Estimated threshold avoidance distance ± Bayesian highest density credible intervals (gray ellipses) for the Lesser Prairie-Chicken, with, for comparative purposes, Bayesian 95% prediction intervals (black lines, with the tick marking the median) and the minimum recorded distance from a feature (x). Results are for centroids of home ranges for the Oklahoma (top) and New Mexico (bottom) study sites. Note the logarithmic scale on the *x*-axis.

Regardless of the density of a feature, we found evidence of avoidance, although estimated threshold distances were shorter in Oklahoma (Table 2, Fig. 5). Estimated thresholds varied across features, often messily. Even so, outbuildings were avoided at a considerable distance (> 1 km) in either state, whereas fences were avoided at a slighter distance (< 100 m) in either state (Fig. 5). State-for-state, estimated thresholds were similar for leks, nests, or home range centroids but were, as logic would dictate, shorter for home range contours (Table 2). Prediction intervals and associated coefficients of variation implied greater uncertainty of distance estimates in Oklahoma irrespective of feature (Appendix S1).

**Table 2:** Results of a Bayesian model to estimate avoidance distance (meters) from various anthropogenic features by the Lesser Prairie-Chicken.

	Threshold	Lower HDI	Upper HDI	Minimum
**Home range—centroid**				
*Oklahoma*				
Road	1095	1085.8	1104.7	138.5
Powerline	253	250.7	255.4	19.6
Oil well	472	469.4	474.9	333.7
Gas well	297	294.4	299.3	26.9
Outbuilding	15,741	14,810.6	16,663.4	1891.0
Fence	518	514.7	522.2	8.0
Railroad track	17,724	17,327.9	18,107.6	7077.3
*New Mexico*				
Road	7052	6859.6	7236.7	1197.7
Powerline	22,901	22,266.8	23,517.5	15,964.7
Oil well	1411	1401.7	1419.9	598.2
Gas well	555	552.3	557.6	554.3
Outbuilding	11,523	11,274.2	11,776.7	8545.0
Fence	729	724.2	733.7	1.6
Private residence	3016	2976.6	3056.6	1123.0
**Home range—outer contour**				
*Oklahoma*				
Road	0	–	–	0.0
Powerline	0	–	–	0.1
Oil well	141	107.6	178.3	7.8
Gas well	0	–	–	1.0
Outbuilding	5026	2940.1	8090.2	1036.5
Fence	0	–	–	0.0
Railroad track	4384	4143.9	4638.1	4048.2
*New Mexico*				
Road	213	211.9	214.4	0.0
Powerline	18,187	17,604.8	18,774.3	10,586.9
Oil well	62	48.1	75.8	17.2
Gas well	184	153.8	213.8	3.2
Outbuilding	9752	9613.2	9885.6	5057.2
Fence	0	–	–	0.0
Private residence	419	413.7	424.1	14.1
**LEK**				
*Oklahoma*				
Road	655	650.6	658.6	611.0
Powerline	391	386.5	395.8	181.5
Oil well	1005	984.6	1025.9	974.0
Gas well	319	315.8	322.5	92.2
Outbuilding	5014	2899.9	7972.8	4327.6
Fence	314	312.3	316.1	45.7
Railroad track	16,851	16,391.1	17,319.7	14,648.4
*New Mexico*				
Road	6945	6859.8	7037.1	2094.4
Powerline	18,537	17,900.91	19,161.3	17,653.8
Oil well	735	732.1	737.6	713.7
Gas well	1075	1067.9	1081.7	994.4
Outbuilding	10,032	9413.8	10,642.5	9818.7
Fence	742	737.9	746.8	193.9
Private residence	3561	3535.5	3587.3	1277.2
**Nest**				
*Oklahoma*				
Road	1250	1237.5	1263.6	150.5
Powerline	293	289.8	295.5	26.7
Oil well	195	192.5	197.6	270.4
Gas well	306	304.9	308.0	91.6
Outbuilding	849	844.4	853.1	772.8
Fence	280	274.0	285.4	11.2
Railroad track	383	362.8	403.8	118.7
*New Mexico*				
Road	7156	7015.3	7296.9	1744.4
Powerline	23,224	22,431.5	24,010.2	14,596.7
Oil well	419	416.4	420.9	364.0
Gas well	426	423.1	429.8	292.3
Outbuilding	11,640	11,331.6	11,963.6	7085.0
Fence	378	376.6	379.9	20.6
Private residence	2361	2336.3	2386.2	850.7

HDI refers to the highest density credible interval, whereas “minimum” is the nearest approach recorded. Dashes indicate that an estimate could not be obtained.

**Figure 6:**
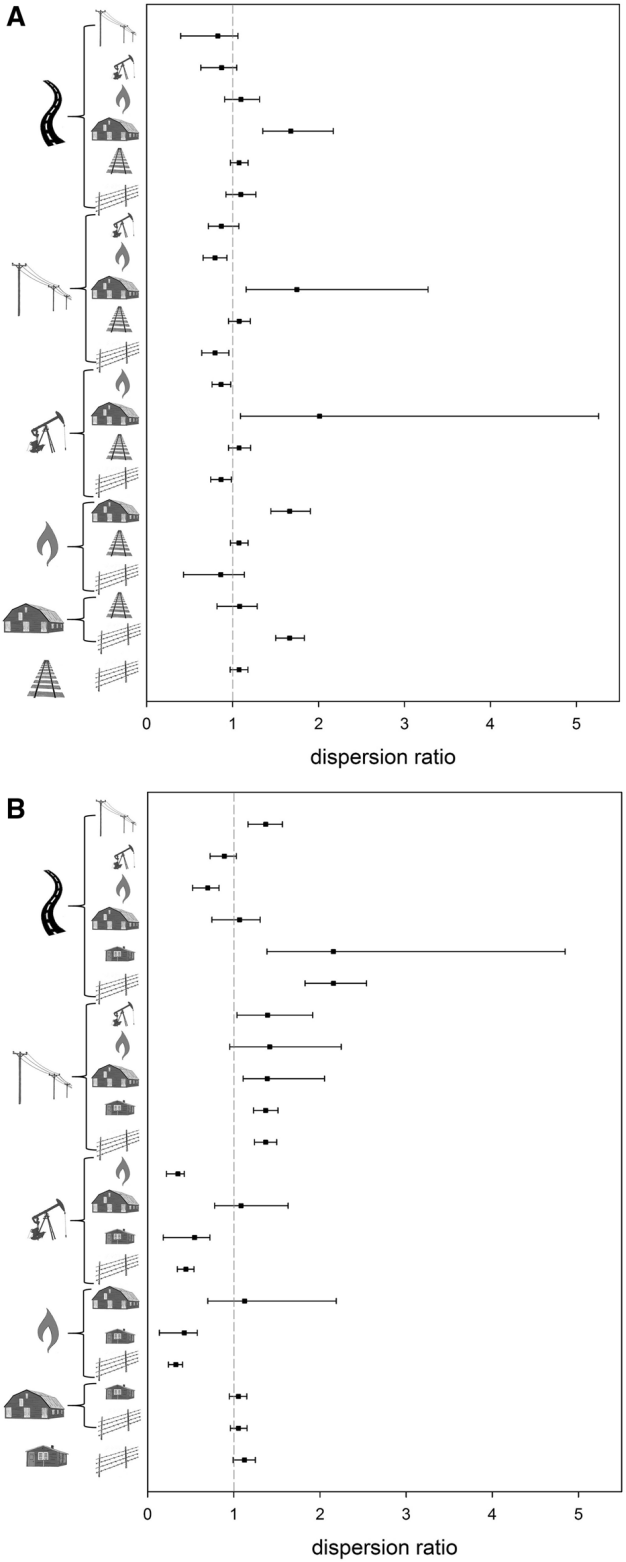
Depiction of clustered (ratio ± Bayesian highest density credible intervals < 1), random (ratio ± CI = 1), or dispersed (ratio ± CI > 1) occurrence of Lesser Prairie-Chickens relative to two features considered jointly (see Fig. 5 for a key to symbols used). Results are for centroids of home ranges for the Oklahoma (top) and New Mexico (bottom) study sites and are assumed symmetrical (i.e., mean distance from, say, fence to road is assumed to be the same as from road to fence) as opposed to a model that allowed for weighted (asymmetrical) ratios.

We found evidence for colocation effects, i.e., where a relationship with one feature might suggest preference or avoidance with another feature. For example, at the Oklahoma study site, grouse consistently avoided outbuildings at a greater distance than other features (Fig. 5), such that estimates of avoidance distances to some features may have been shorter than if outbuildings were removed from the landscape; i.e., the dispersion ratio was consistently > 1.0, implying strong avoidance of one feature (outbuildings) relative to other features. Conversely, gas wells and fences were viewed as more benign, all else being equal (Fig. 6). The latter pattern held in New Mexico, too, where in some circumstances oil wells appeared to be “lesser evils” (Fig. 6). At that study site, though, grouse avoided powerlines (in particular) and roads at a greater distance than they did in Oklahoma (Fig. 5), such that higher dispersion ratios were estimated when response to a more “severe” feature (e.g., a road) was compared to a more “benign” feature (e.g., a fence).

## Discussion

### Avoidance behavior

Many organisms are reported to avoid anthropogenic features^9,10,24–26^, yet the “unintended but potentially profound consequences of infrastructure on animals remain poorly understood”^27^. The nature of avoidance exacerbates its study because observed absence of use must be relied upon as an indication of putative avoidance behavior; hence, we analyzed binned data of habitat use to elucidate feature avoidance. In doing so, we add to evidence that the Lesser Prairie-Chicken avoids various man-made features^9,28–30^, but our analyses go further because they provide estimates of avoidance distances. A quantified animal behavior such as avoidance distance can inform conservation planning, concerns about transferability among sites and species notwithstanding^31^. Such concerns may have merit in this system in light of distinct differences in avoidance distances between our two study sites, which differ fundamentally in both land tenure (see “Methods”: Data) and floristic composition and structure^11,12^.

Leveraging Markov chain Monte Carlo to approximate a posterior habitat preference distribution for models considering animals’ exposure to dis-amenity or negative cues constitutes a novel way to describe influences that lead to avoidance. Research that used animal locations to infer habitat use has informed spatial ecology, movement ecology, and conservation planning. Following the early logic of bounding extent methods that examined habitat availability and use from known animal locations, improvements to understanding habitat use have incorporated approaches from computational geometry^32^, probabilistic approaches^33,34^, simulation^35–37^, and methods adapted from time geography^38–40^ in their estimation of utilization distributions. Methods that combine and connect ideas among these themes are emergent, too^41^. Combination of the action and effect of *preference* in these estimates generally is limited. Modeling behavioral influences on the expression of habitat avoidance represents an unexplored and promising avenue for growth in this pursuit.

Our analyses allowed us to reject our first hypothesis: rather than distribute themselves randomly across the landscape, Lesser Prairie-Chickens avoid anthropogenic features. We likewise were able to reject our second hypothesis: anthropogenic features are not avoided uniformly. Instead, some features were avoided at a greater distance than others, even when accounting for differences in feature density, a pattern that held in both study areas and whether we considered home range centroid, lek location, or nest placement. Features avoided the most tended to be tall, imposing, or noisy, such as powerlines, outbuildings, and roads, whereas those avoided the least tended to be small and static, such as gas wells and fences, a result that lent support to our third hypothesis. Even so, this result is complicated by apparent covariation in avoidance, corresponding to our fourth hypothesis, which is not mutually exclusive of the third. We suggest that strong avoidance of large, imposing features reduced estimates of avoidance to other, less imposing features.

Our specific findings contradict those reported in a review paper on avoidance behavior in grouse^10^. Across 8–9 species of grouse, including the Lesser Prairie-Chicken, they “found that oil and gas structures had the greatest negative effect on displacement behaviour”, yet our data for a single species indicated that oil well and gas wells had among the shortest avoidance distances, and avoidance distances were less than the median distances from random points to the features, indicating that the threshold occurred well before feature density saturated on the landscape. Likewise, the review^10^ reported a low effect of displacement from powerlines and outbuildings, yet we found that outbuildings, especially, had among the highest avoidance thresholds at our two study sites. Our results correspond better to those of two subsequent studies focused on the Lesser Prairie-Chicken^29,30^, which reported strong avoidance of tall features such as powerlines and wind turbines; e.g., “[d]istance to powerline [was] the single most consistent variable negatively affecting nest placement,” and oil wells had no effect on placement of home ranges or nests^29^. We found that grouse avoided oil wells, as did one study^29^ when data were pooled, and both that study and ours found that avoidance of oil wells was of a smaller magnitude (i.e., shorter distance) than avoidance of other features. Across all studies, including the important meta-analysis^10^, it is clear that grouse avoid man-made features, with avoidance distance varying with feature type.

We caution that our estimates of avoidance distance, as with those in many studies, are based on best available data but nonetheless may suffer from exactly where the study area was sited. Perhaps a study area was selected that had numerous long-standing leks but in or near which no development had occurred. Compared to a study area embedded in an area of intensive land use, estimates of avoidance distance would necessarily be higher. We are confident that our methods elucidate *relative* avoidance distances well, but if we assume an estimable parameter for avoidance distance exists, then such estimates are highly uncertain (Fig. 5). After all, apparent avoidance could result from human preference to build in certain parts of a landscape (e.g., in valley floors) or a legacy of haphazard settlement patterns. Our model did not incorporate as a covariate variation across the landscape in vegetation or geography, because we only sampled two non-overlapping ecoregions for a species with extremely specific structural preferences. Nonetheless, our model could be modified readily to accommodate such covariates in other study systems that include more types of land cover, ecoregions in common between study sites, or in species with a wider range of remotely detectable vegetation differences (or with more local vegetation data obtained). Short of controlled experiments, which are nearly impossible in studies of avoidance of large features, the ideal is a before-after/control-impact (BACI) design^42^. Even so, comparisons between areas with striking differences in feature density, as in our system (Table 1), are valuable, too, in the same way that the comparative method is valuable in evolutionary studies.

### Habitat selection and avoidance

Our analytic approach points toward a means to incorporate avoidance behavior directly into studies of habitat selection. At root, we focus on providing a model of animal perception, an intrinsic aspect of an organism that governs how cues are assessed to judge habitat suitability, the very substance of habitat preference (Figs. 1, 2). The probability distribution^2^ of preference is not fixed but is shaped by fitness consequences of habitat use. It is a feedback loop: observed habitat use by individuals is a result of preference and pressures and context that shape selection, with use in turn affecting preference visible in populations. Habitat selection is itself a complex and temporally dynamic process driven initially by preference but shaped by various extrinsic factors, from competition to predation and from disturbance to availability.

It is imperative that we begin to link sensory ecology, including avoidance, to basic aspects of habitat occupancy and associated aspects of conservation biology^43^. Yet where an organism settles in space is only part of its overall life history. There are consequences to habitat use, good or ill. Such fitness consequences—whether effects to mate acquisition, reproductive output, or survival—will inform and update the prior to the extent that perception of and action on cues ultimately are heritable. In this light, an organism’s small and routine daily decisions manifest over evolutionary time.

## Supplementary Information

Supplementary Table.
