# Supplementary material for: Forging a Bayesian link between habitat selection and avoidance behavior in a grassland grouse
Source: Sci Rep. 2021 Feb 2;11:2791. doi: 10.1038/s41598-021-82500-0 (PMC7854595; doi:10.1038/s41598-021-82500-0)
Supplement: Supplementary file 1 — Supplementary Table. [file 41598_2021_82500_MOESM1_ESM.pdf]

Supplementary information

Forging a Bayesian link between habitat selection and avoidance behavior in a grassland grouse

Michael A. Patten, Alexandra A. Barnard, Claire M. Curry, Henry Dang, and Rebecca W. Loraamm

Appendix S1. Bayesian prediction intervals and coefficients of variation (CV) for Lesser Prairie-Chicken (*Tympanuchus pallidicinctus*) occurrence near anthropogenic features. Note the consistently higher variability for the Oklahoma data set. HDI refers to the highest density credible interval.

	median	lower HDI	upper HDI	CV
		**HOME RANGE – CENTROID**		
Oklahoma				
road	2491	110	11,342	0.564
powerline	2636	45	17,495	0.559
oil well	3996	475	12,539	0.475
gas well	596	49	2159	0.555
outbuilding	8233	3719	14,891	0.324
fence	433	1	4104	0.686
railroad	18,787	11,360	28,706	0.228
New Mexico				
road	4844	1779	9624	0.324
powerline	20,776	16,073	25,780	0.118
oil well	2393	950	4588	0.358
gas well	2256	616	5201	0.467
outbuilding	11,665	9219	14,497	0.113
fence	587	30	2611	0.514
residence	2798	1120	5195	0.346
		**HOME RANGE – OUTER CONTOUR**		
Oklahoma				
road	380	0	>50 km	1.055
powerline	586	0	>50 km	0.966
oil well	2286	8	20,385	0.684
gas well	100	0	893	1.666
outbuilding	6891	2659	13,474	0.356
fence	4	0	992	3.069
railroad	17,437	9441	27,745	0.244
New Mexico				
road	2522	5	27,621	0.512
powerline	19,028	14,079	24,943	0.139

oil well	1015	39	4750	0.639
gas well	751	3	6777	0.909
outbuilding	10,247	7501	13,397	0.137
fence	2	0	93	2.889
residence	1302	61	5019	0.589
		**LEK**		
Oklahoma				
road	2488	160	9610	0.565
powerline	2717	31	19,220	0.607
oil well	4517	712	12,875	0.484
gas well	683	95	2046	0.547
outbuilding	7817	3245	14,825	0.362
fence	389	2	3440	0.710
railroad	19,949	11,212	30,687	0.288
New Mexico				
road	5098	2152	9426	0.289
powerline	20,634	16,344	25,268	0.110
oil well	2341	1032	4364	0.282
gas well	2458	771	5184	0.477
outbuilding	12,162	9294	15,321	0.127
fence	723	227	1557	0.361
residence	2965	1164	5563	0.322
		**NEST**		
Oklahoma				
road	2713	130	12,121	0.552
powerline	2823	20	22,048	0.585
oil well	3655	225	14,886	0.521
gas well	610	67	1959	0.597
outbuilding	7807	2883	15,241	0.312
fence	455	2	4407	0.696
railroad	17,225	3584	42,828	0.294
New Mexico				
road	4506	1449	9528	0.366
powerline	21,200	16,102	27,121	0.125
oil well	2537	773	5329	0.387
gas well	2362	479	5911	0.470
outbuilding	11,613	8818	14,985	0.130
fence	472	14	2259	0.589
residence	2530	984	4815	0.364
